# Obesity and its association with self-efficacy and metabolic risk factors by region of birth among 40-year-olds participating in the Swedish targeted health dialogues

**DOI:** 10.1016/j.pmedr.2024.102845

**Published:** 2024-08-05

**Authors:** Beata Borgström Bolmsjö, Simona Chiarappa, Emelie Stenman, Anton Grundberg, Kristina Sundquist

**Affiliations:** a Center for Primary Health Care Research, Department of Clinical Sciences Malmö, Lund University, Malmö, Sweden; b University Clinic Primary Care Skåne, Region Skåne, Sweden; c University of Gothenburg, School of Public Health and Community Medicine, Institute of Medicine, Sahlgrenska Academy, Sweden

**Keywords:** Immigration, Lifestyle intervention program, Metabolic risk factors, Obesity, Risk of cardiovascular disease, Self-efficacy

## Abstract

**Objective:**

Targeted Health Dialogues (THD) is a public health intervention program that aims at preventing cardiovascular disease. THDs were implemented in the Swedish region Scania, in 2020, with the novelty of being conducted in a metropolitan area with a multiethnic population.

This study investigated the prevalence of obesity among 40-year-old THD participants in Scania by region of birth, and its associations with self-efficacy and additional metabolic risk factors.

**Methods:**

Cross-sectional data were retrieved from measurements in the THDs. Study participants included 1831 40-year-olds. Differences in characteristics by region of birth were assessed using chi-squared and ANOVA tests. The associations between overweight, obesity, and high waist-hip ratio (WHR) and self-efficacy and metabolic risk factors (blood pressure, LDL cholesterol, plasma glucose) were assessed using binominal and ordinal logistic regression, adjusted for sex and education and tested for interactions by region of birth.

**Results:**

35.1 % of the participants were overweight (BMI 25–29.9), and 18.7 % were obese (BMI ≥30) with the highest levels among participants born outside Sweden (p = 0.005). Abdominal obesity was also more prevalent among participants born outside Sweden (p = 0.002). Obesity was associated with increased odds of having low self-efficacy (OR per BMI-level: 1.48 (CI 1.24–1.76) and additional metabolic risk factors. No interactions with region of birth were detected.

**Conclusions:**

The prevalence of obesity differed between region of birth and obesity was associated with having low self-efficacy. These findings underline the need to customise lifestyle interventions in a multiethnic population to increase health equity.

## 1 Introduction

Cardiovascular disease (CVD) is a major cause of morbidity and mortality in industrialised countries (Perk et al., 2012), the leading cause of premature death in Europe and the most common somatic disease behind loss of productivity (Scottish Intercollegiate Guidelines Network (SIGN), 2007). Therefore, it is important to identify CVD risk factors early in life in order to be able to treat them before CVD occurs.

Overweight and abdominal obesity (high waist-hip ratio, WHR) are associated with higher risks of CVD, other chronic diseases, and mortality. Since 1975 the prevalence of obesity has increased steeply in the whole world (World Health Organization (WHO), 2018), thus making obesity a global health issue. Sweden is no exception, with an alarming increase in the prevalence of obesity among all age groups (Neovius et al., 2006).

Self-efficacy is a self-reported measure that indicates the perception that a person has of his/her ability to make a change (Bandura, 1977) and a high self-efficacy could therefore support a behavioural change. Measuring self-efficacy can be useful in treating chronic conditions, where self-management may often be vital. High self-efficacy is hypothesised to create better health outcomes and help reduce the health care burden (Marks et al., 2005). It has also been suggested to be a predictor of weight control (Teixeira et al., 2015). Obesity has in itself a multifactorial aetiology and is often difficult to treat effectively (Tutor et al., 2023). The subsequent metabolic risk (Park et al., 2003, Redon et al., 2008) may however be possible to decrease by a generally improved lifestyle, and this may be facilitated by a belief in your own ability to make a change, i.e., high self-efficacy.

The Swedish screening and intervention program Targeted Health Dialogues (THD) has been implemented in different provinces of Sweden since the 1980 s to increase awareness of risk factors for CVD and to encourage healthy lifestyle choices at the primary care level (Lingfors et al., 2009). It has been shown, with low to moderate levels of evidence, to reduce cardiovascular mortality (The Swedish Association of Local Authorities and Regions, 2022) and to be cost-effective (Lindholm et al., 2018). In the southernmost province of Sweden, Scania, the THDs were implemented in for the first time in 2020 targeting initially the 40-year-olds. Scania is characterised by a multiethnic population with around 1.4 million inhabitants. During recent decades, Sweden has had an increase in international immigration. According to Statistics Sweden (2021), Sweden has a population of around 10 million inhabitants with about two million being foreign-born. In Scania, around 23 % of inhabitants are foreign-born, stemming from 179 different countries (Region Skåne, 2023).

Thus, the migrant population in Scania adequately mirrors the Swedish migration pattern, which has been characterised in recent decades by a shift from labour migrants from other European countries to an increase of refugees from non-European countries.

It is well-known that migration itself can increase the vulnerability to ill-health after a first period of 5–10 years when migrants tend to have better health than the host population, the so-called Healthy migrant effect (Helgesson et al., 2018). However, the Healthy migrant effect has not been clearly observed in migrants in Sweden and may differ between labour migrants and refugees (Helgesson et al., 2018). Studies in a Swedish region with a high proportion of non-European immigrants are therefore relevant, giving us the opportunity to study health status in relation to region of birth.

The aim of this study was to describe the prevalence of overweight, obesity, and abdominal obesity by region of birth in 40-year-olds participating in the THDs in Scania. Since self-efficacy has been suggested to influence lifestyle and weight control, we also wanted to examine possible associations between self-efficacy and high BMI/abdominal obesity, and if the associations differed by region of birth. Furthermore, we wanted to examine associations between high BMI/abdominal obesity and other metabolic risk factors (elevated blood pressure, LDL cholesterol, and plasma glucose) in our population, also by region of birth.

## 2 Methods

### 2.1 Design and study population

The data were cross-sectional and retrieved from the measurements of participants in the THDs in Scania during 2021 and the first half of 2022. The THDs were conducted in primary care settings by specially trained personnel and health dialogue coaches from the primary health care centres. The health dialogue coaches were registered professionals such as nurses, dieticians, occupational- or physiotherapists or physicians, who went through a three-day course in motivational interviewing and a two-day course in the THD method.

All people living in Scania are registered at a specific primary health care centre, of their own choice. At the time of data collection, 99 of the province’s 170 primary healthcare centres had started with the regionwide intervention THDs. Those who turned 40 years of age in 2021 and in the first half of 2022, and belonged to one of these 99 health care centres, were invited to take part in a THD. Furthermore, all participants who chose to participate in the THDs were asked if their data could be used for further research projects. If so, they provided written consent for the research project.

### 2.2 Measurements

Before the THD, participants filled in a detailed questionnaire regarding their health, health behaviours, and social factors (including self-efficacy). Thereafter, they visited their healthcare centre for a collection of anthropometric measures and laboratory data such as blood pressure, BMI, WHR, LDL-cholesterol and fasting blood glucose. Collected data from the questionnaire and measurements were used as a basis for the THD about a week later.

Overweight was defined as a BMI (kg/m^2^) ≥25 kg/m^2^ and obesity as a BMI ≥30 kg/m^2^, according to the WHO (World Health Organization (WHO), 2018). High waist–hip ratio was defined as ≥0.85 for women and ≥0.90 for men according to the WHO definition of abdominal obesity in metabolic syndrome (World Health Organization (WHO), 2006).

Self-efficacy was assessed by a question adapted from previous literature (Pearlin and Schooler, 1978): “To what degree do you agree with the statement ‘I believe that I can influence my health’?”. The possible answers were: 1. Agree, 2. Partly agree, 3. Partly disagree, 4. Disagree. For the analyses, we dichotomised the answers into two groups. The participants that agreed (response alternative 1) with the statement “I believe that I can influence my health” were categorised as having high self-efficacy and the participants with response alternatives 2–4 were categorised as having low self-efficacy.

Blood pressure was measured in a sitting position after resting for five minutes. It was measured in both arms and two times with the mean value registered. High blood pressure was defined as systolic blood pressure ≥140 mmHg and/or diastolic blood pressure ≥90 mmHg according to European guidelines (Williams et al., 2018). High cholesterol was defined as LDL-cholesterol ≥5 mmol/L, according to international guidelines (Grundy et al., 2019). Regarding glucose, a cut-off value of ≥6.1 mmol/L was set to define impaired fasting glucose and a cut-off ≥7 to define suspicion of diabetes, according to the WHO definitions from 2006 (World Health Organization (WHO), 2006). Sex of the participants was collected automatically from the social security number in the medical records, which indicates sex at birth (or sex after complete gender transition).

Education was defined into three categories (low: ≤9 years of school, middle: 10–12 years of school and high: >12 years of school). Region of birth was defined as Swedish-born or foreign-born; the latter was further divided into other European countries and non-European countries. Time spent in Sweden for the individuals born outside Sweden was dichotomised to ≤10 years and >10 years.

### 2.3 Data analysis

Differences in patient characteristics based on region of birth were tested using chi-squared tests for categorical variables and ANOVA tests for continuous variables. The tests were also adjusted, using logistic regression, for level of education and sex (except for WHR, which in itself is already adjusted for sex). Also differences in obesity indices, BMI and WHR, and self-efficacy for the immigrant groups, based on their time spent living in Sweden were calculated with chi-squared tests. To assess the associations between the obesity indices and other risk factors, odds ratios (OR) with 95 % confidence intervals (CI) were estimated using binomial logistic regression. The associations between self-efficacy and the obesity indices, each as separate outcomes, were also estimated using logistic regression. Specifically, binomial logistic regression was used modelling WHR as outcome, and ordinal logistic regression (proportional odds model) when using BMI as the outcome. Ordinal logistic regression allows the outcome to have more than two categories, with an ordered structure. We were thus able to analyse BMI as an outcome resulting in an OR, in a similar manner as the other analyses of association, while keeping the clinically relevant cut-off levels at both ≥25 (overweight) and ≥30 (obese). The assumption of proportional odds was tested using a Brant test.

All ORs were estimated after adjusting for sex (except for WHR) and level of education and stratifying by region of birth. We also tested for interactions with region of birth by adding an interaction term (instead of stratifying), and by using a likelihood ratio test. All analyses were complete case analyses; thus, missing data were not handled specifically. A p-value of <0.05 was considered statistically significant. All statistical analyses were performed in R, version 4.2.1 (R Core Team. R, 2022).

### 2.4 Ethical approval and consent to participate

The study has been carried out in accordance with The Code of Ethics of the World Medical Association (Declaration of Helsinki) and was approved by the Swedish Ethical Review Authority, with registration number 2020–02689 with later amendments. A written informed consent form was provided by all participants in the THD who agreed to take part in the research project.

## 3 Results

In total, 1831 (1010 female [55.2 %] and 821 male [44.8 %]) participants completed both the health questionnaire and the health dialogue, and also gave consent to participate in the research project. The study population consisted of 68.4 % Swedish-born participants, 14.7 % born in other European countries and 16.9 % born outside Europe. There were significant differences in educational level between the groups. The foreign-born participants, independent of their origin, had a lower level of education compared to the Swedish-born. In particular, 18.1 % of the 40-year-olds from non-European countries had nine years or less of education (p < 0.001) versus only 2.9 % of the Swedish-born (Table 1).

**Table 1:** Descriptive characteristics of the participants in Targeted Health Dialogues in Scania, Sweden, Jan 2021- June 2022.

	Overall	Sweden	Other European country	Non-European country	p-value a
**Region of birth, n (%)**	1831 (1 0 0)	1252 (68.4)	270 (14.7)	309 (16.9)	<0.001
**Sex, n (%)**					0.86
Male	821 (44.8)	561 (44.8)	118 (43.7)	142 (46.0)	
Female	1010 (55.2)	691 (55.2)	152 (56.3)	167 (54.0)	
**Level of education, n (%)**					<0.001
≤9 years	107 (5.8)	36 (2.9)	15 (5.6)	56 (18.1)	
10–12 years	602 (32.9)	415 (33.1)	101 (37.4)	86 (27.8)	
>12 years	1120 (61.2)	801 (64.0)	154 (57.0)	165 (53.4)	
Missing	2 (0.1)	0 (0)	0 (0)	2 (0.6)	
**BMI, mean (SD)**	26.28 (4.77)	26.11 (4.82)	26.52 (4.74)	26.74 (4.56)	0.072
**BMI, n (%)**					0.005
<25	842 (46.0)	605 (48.4)	120 (44.4)	117 (37.9)	
25–29.9	643 (35.1)	431 (34.4)	88 (32.6)	124 (40.1)	
≥30	342 (18.7)	213 (17.0)	61 (22.6)	68 (22.0)	
Missing	4 (0.2)	3 (0.2)	1 (0.4)	0 (0)	
**Waist-hip ratio, n (%)**					0.002
Normal b	1089 (59.5)	782 (62.5)	147 (54.4)	160 (51.8)	
High c	718 (39.2)	460 (36.7)	117 (43.3)	141 (45.6)	
Missing	24 (1.3)	10 (0.8)	6 (2.2)	8 (2.6)	
**Self-efficacy, n (%)**					0.042
High	963 (52.6)	685 (54.7)	128 (47.4)	150 (48.5)	
Low	860 (47.0)	565 (45.1)	140 (51.9)	155 (50.2)	
Missing	8 (0.4)	2 (0.2)	2 (0.7)	4 (1.3)	
**Systolic blood pressure, mean (SD)**	121.8 (13.4)	122.7 (13.0)	121.5 (13.5)	118.3 (14.2)	<0.001
**Diastolic blood pressure, mean (SD)**	78.61 (10.18)	78.63 (9.82)	79.15 (11.26)	78.07 (10.63)	0.44
**Blood pressure, n (%)**					0.048
≤139/≤89 mmHg	1488 (81.3)	1004 (80.2)	217 (80.4)	267 (86.4)	
≥140/≥90 mmHg	339 (18.5)	245 (19.6)	52 (19.2)	42 (13.6)	
Missing	4 (0.2)	3 (0.2)	1 (0.4)	0 (0)	
**LDL, mean (SD)**	3.29 (0.95)	3.25 (0.94)	3.37 (0.96)	3.38 (0.96)	0.026
**LDL Cholesterol, n (%)**					0.41
<5 mmol/l	1730 (94.5)	1189 (95.0)	253 (93.7)	288 (93.2)	
≥5 mmol/l	90 (4.9)	56 (4.5)	15 (5.6)	19 (6.1)	
Missing	11 (0.6)	7 (0.6)	2 (0.7)	2 (0.6)	
**Fasting-plasma-glucose, mean (SD)**	5.44 (0.79)	5.42 (0.84)	5.48 (0.67)	5.48 (0.64)	0.29
**Fasting-plasma-glucose, n (%)**					0.34
≤6 mmol/l	1647 (90.0)	1139 (91.0)	237 (87.8)	271 (87.7)	
6.1–6.9 mmol/l	133 (7.3)	81 (6.5)	23 (8.6)	29 (9.4)	
≥7 mmol/l	35 (1.9)	22 (1.8)	6 (2.2)	7 (2.7)	
Missing	16 (0.9)	10 (0.8)	4 (1.5)	2 (0.6)	

a Difference between region of birth tested by chi-square-test for categorical variables (not including missing values) and ANOVA test for continuous variables.

b ≤0.84 for women/≤0.89 for men.

c ≥0.85 for women/≥0.90 for men.

### 3.1 BMI and waist-hip ratio by region of birth

**Fig. 1:**
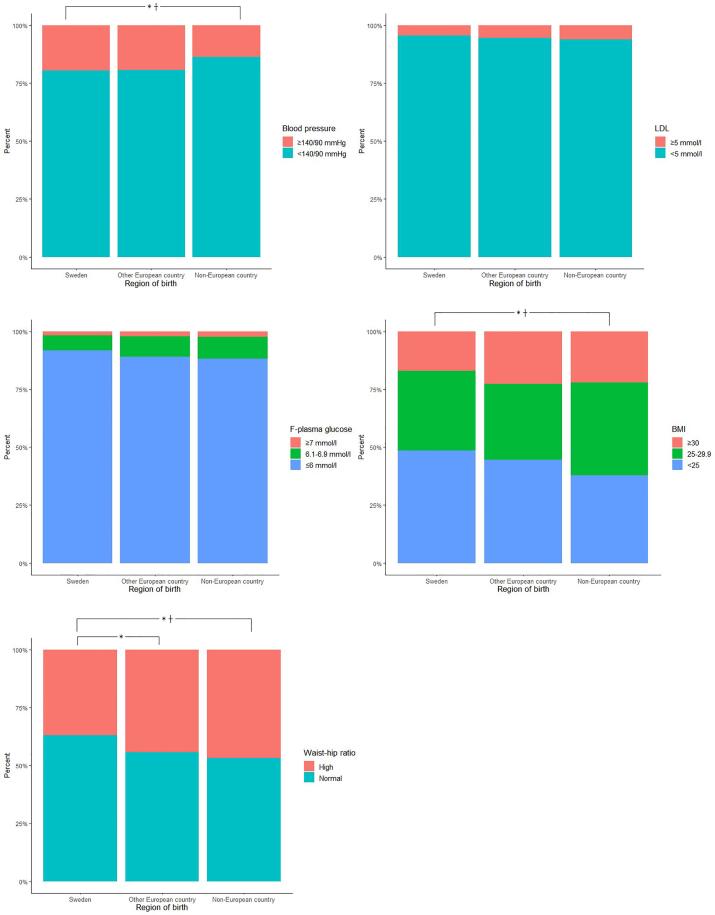
**Metabolic risk factors broken down by region of birth for the participants in Targeted Health Dialogues in Scania, Sweden, Jan 2021- June 2022.** * p-value < 0.05 when unadjusted, ┼ p-value < 0.05 when adjusted for sex and level of education (WHR is adjusted only for level of education).

In the total study population, 46 % had a normal weight (BMI<25), 35.1 % were overweight (BMI 25–29.9 %), and 18.7 % were obese (BMI≥30). However, there were significant differences in BMI (p = 0.005) and WHR (p = 0.002) between participants by different regions of birth. Participants born outside of Sweden was the group with the highest proportion of obesity (Table 1, Fig. 1).

In the total study population, there were 718 (39.2 %) participants with abdominal obesity and the highest proportion, 45.6 %, was found in the group born outside of Europe. Mean BMI (continuous variable) in the total population did not differ significantly by country of birth (Table 1). For the participants with origin outside of Sweden, time spent in Sweden did not seem to affect the BMI nor WHR (Table 2).

**Table 2:** Distribution of BMI, WHR and self-efficacy, by time spent living in Sweden for foreign born participants in Targeted Health Dialogues in Scania, Jan 2021- June 2022.

	Time spent in Sweden, n (%)		p-value^a^
	≤10 years	>10 years	
Other European country			
**BMI**			0.61
<25	37 (49.3)	83 (42.8)	
25–29.9	23 (30.7)	65 (33.5)	
≥30	15 (20.0)	46 (23.7)	
**WHR**			0.76
Normal^b^	39 (54.2)	108 (56.2)	
High^c^	33 (45.8)	84 (43.8)	
**Self-efficacy**			0.85
High	37 (48.7)	91 (47.4)	
Low	39 (51.3)	101 (52.6)	

Non-European country			
**BMI**			0.93
<25	47 (37.3)	68 (38.0)	
25–29.9	50 (39.7)	73 (40.8)	
≥30	29 (23.0)	38 (21.2)	
**WHR**			0.19
Normal	73 (57.9)	86 (50.3)	
High	53 (42.1)	85 (49.7)	
**Self-efficacy**			0.35
High	65 (52.4)	83 (46.9)	
Low	59 (47.6)	94 (53.1)	

a Proportional difference by time spent in Sweden tested by chi-square-test.

b ≤0.84 for women/≤0.89 for men.

c ≥0.85 for women/≥0.90 for men.

### 3.2 Self-efficacy by region of birth

In the total study population, 47 % of participants reported low self-efficacy. The group that was born in other European countries had the highest proportion of low self-efficacy (51.9 %) (Table 1). Self-efficacy did not differ significantly depending on time spent in Sweden (Table 2).

### 3.3 Metabolic risk factors by region of birth

In the analyses of categorical variables, only blood pressure differed between the different groups, with a significantly higher proportion of high blood pressure in the Swedish-born population compared to the non-European-born. In the analyses based on continuous variables, the systolic blood pressure was significantly higher among Swedish-born compared to foreign-born participants. On the other hand, mean level of LDL-cholesterol was significantly higher among foreign-born compared to Swedish-born participants (Table 1, Fig. 1).

### 3.4 Associations between high BMI/abdominal obesity and self-efficacy by region of birth

**Fig. 2:**
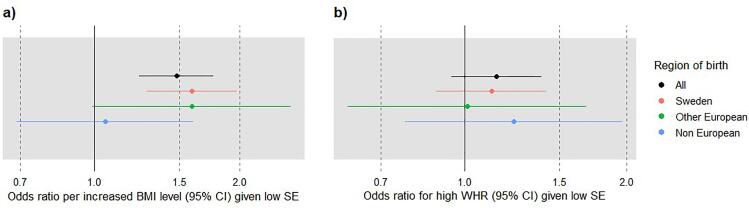
**Odds ratio per increased BMI level and for abdominal obesity among the participants in Targeted Health Dialogues in Scania, Sweden, Jan 2021- June 2022, with low self-efficacy (SE) (ref. high self-efficacy)**. Data stratified by region of birth and adjusted for sex (BMI) and level of education (BMI and WHR). **a)** Odds ratio per increased BMI level (outcome), BMI is categorised with three levels: <25, 25–29.9 and ≥30. OR is estimated using ordinal logistic regression. **b)** Odds ratio for abdominal obesity (outcome), abdominal obesity is dichotomised with cut-off at WHR ≥0.9(M)/0.85(F). OR is estimated using binary logistic regression.

Low self-efficacy was associated with high BMI in the total population and in the Swedish-born participants, after adjustment for sex and level of education (ORs in the total population 1.48 (CI 1.21; 1.76) per increased BMI-level; OR 1.15 (CI 0.94; 1.39) for abdominal obesity) (Fig. 2). There were no significant associations between low self-efficacy and high BMI/WHR in the foreign-born groups, but we could not detect any differential effects of self-efficacy on obesity based on region of birth in the interaction analysis (data not shown).

### 3.5 Associations between obesity, overweight and metabolic risk factors by region of birth

**Fig. 3:**
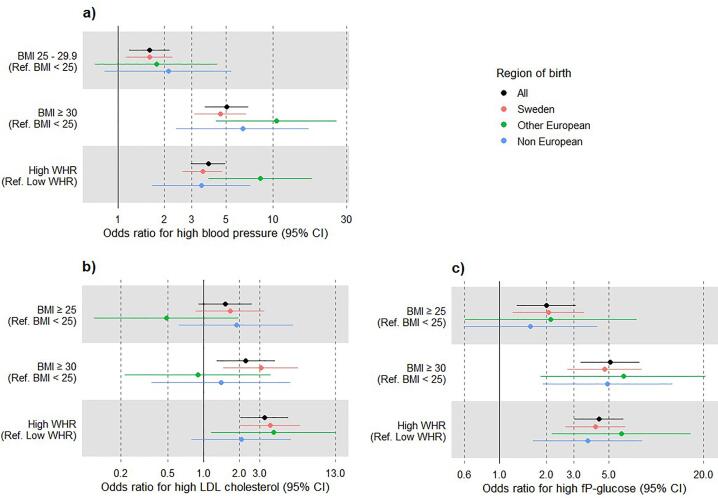
**Odds ratio for high blood pressure, LDL cholesterol and fP-glucose among the participants in Targeted Health Dialogues in Scania, Sweden, Jan 2021- June 2022, with high BMI and WHR.** Data stratified by region of birth and adjusted for sex (BMI) and level of education (BMI and WHR). **a)** Odds ratio for high blood pressure, blood pressure is dichotomised with cut-off at ≥140/90 mmHg. **b)** Odds ratio for high LDL cholesterol, LDL cholesterol is dichotomised with cut-off at ≥5 mmol/l. **c)** Odds ratio for high fP-glucose, fP-glucose is dichotomised with cut-off at >6 mmol/l. All ORs are estimated using binomial logistic regression. WHR is dichotomised with cut-off at ≥0.9(M)/0.85(F).

Participants with obesity (BMI ≥30) were more likely than participants with normal weight (BMI <25) to have high blood pressure, high LDL, as well as high fasting glucose (Fig. 3). For instance, obesity was associated with a five times higher likelihood of having high blood pressure compared to the normal weight population when adjusted for sex and level of education. Likewise, abdominal obesity was strongly associated with having other metabolic risk factors. When stratified by region of birth, participants born outside Sweden did not show significant ORs regarding the association between overweight, obesity, abdominal obesity and high LDL-cholesterol (with an exception for abdominal obesity and high LDL-cholesterol in participants from European countries outside Sweden), nor between overweight and high fasting plasma glucose as well as overweight and high blood pressure (Fig. 3). There was, however, no significant interactions between the obesity indices and region of birth (data not shown).

## 4 Discussion

This study showed that foreign-born participants, in general, suffered from overweight/obesity and low self-efficacy to a higher degree compared to Swedish-born participants. Furthermore, obesity was associated with increased odds of having low self-efficacy and additional metabolic risk factors. Neither the level of BMI, WHR nor self-efficacy were affected by the time spent in Sweden for the groups with a foreign background.

There were no interaction effects by region of birth between obesity and having low self-efficacy, nor between obesity and additional metabolic risk factors by region of birth.

Studies have demonstrated that overweight and abdominal obesity are major predictors of the metabolic syndrome and other cardiovascular risk factors (Park et al., 2003, Redon et al., 2008). Although the risk of developing the metabolic syndrome was evident in the obese population, it is notable that the risk increased steeply already in the overweight population (Park et al., 2003). As our study showed that a remarkably high proportion of the 40-year-olds in Scania were already overweight, obese or had abdominal obesity, these factors need to be addressed to prevent future development of the metabolic syndrome and CVD. Almost 19 % of the individuals in our study population had obesity. In the total Swedish adult population, the corresponding figure is around 16 % (The Public Health Agency of Sweden, 2024), and globally around 13 %. However, the current situation in Sweden remains significantly less severe compared to the United States who has an obesity prevalence of >40 % (Tutor et al., 2023).

Results from previous studies have shown an influence of migration and region of birth on BMI (Pudaric et al., 2000, Sundquist and Winkleby, 2000). Our findings of a higher proportion of obesity and abdominal obesity in the group of foreign-born compared to Swedish-born participants are consistent with findings from other studies (Bennet et al., 2011, Sundquist and Johansson, 1998), showing such disparities already at the relatively young age of 40 years.

Studies have shown that immigrants often face a downward trend in social mobility, which can negatively impact their health as well as their self-efficacy (Ramos et al., 2018). In our study, 47 % of the total study population reported low self-efficacy, with a higher proportion of low self-efficacy among the participants born outside of Sweden. As migration could lead to adverse changes in lifestyle, such as decreased physical activity and impaired dietary habits, that may further increase the risk of ill health (Rosenthal et al., 2022), it may be important to find ways to increase self-efficacy in these groups for more effective health interventions.

Prior studies have shown that obesity is related to low self-efficacy (Teufel et al., 2013). This also was seen in our study looking at all of the study population. The test for interactions could not detect any differential effects of self-efficacy on obesity/overweight or abdominal obesity by region of birth. Still, the potential impact of cultural differences on how self-efficacy relates to obesity needs to be further examined for the development of population-based programs tailored to heterogeneous populations. It should be noted that the potential results of the THDs may depend on a belief in onés own ability to influence one’s health, i.e. self-efficacy, since the focus is on improving lifestyle to prevent diseases. Thus, more knowledge of self-efficacy and its association with health outcomes in different groups is crucial for the implementation of the THDs.

It is also notable that the prevalence of high blood pressure was higher in the Swedish-born group, despite their lower weight. This is also in line with previous studies (Bennet et al., 2011). In the present study population, we could not detect any interaction effects between overweight/obesity and region of birth in the associations with metabolic risk factors, but such effects may emerge later in life, which may need to be studied further. Regardless of this, the differences seen in metabolic risk factors depending on region of birth underline the importance of adapting lifestyle and CVD risk interventions in a more tailored manner based on the population in focus.

A strength of the present study is that the THDs were implemented according to a well-established protocol (Lingfors and Persson, 2019) with educated study personnel who conducted objective measurements of parameters including BMI, WHR and other metabolic risk factors. In addition, the method has been widely used and validated in Sweden since the 1980s.

A limitation of the study was the lack of knowledge of the specific countries of birth, which restricted the possibility of examining specific country groups. Yet another limitation was that, at the time when we retrieved the research data, 99 out of ca 170 healthcare centres had implemented THDs. This may have affected the external validity if the populations in these healthcare centres differed from the whole region. However, the method was introduced in the entire region simultaneously, with a mix of public and private healthcare centres (both tax financed).

### 4.1 Conclusions

The results of this study have demonstrated that in the relatively young age group of 40-year-olds, there is a high prevalence of overweight/obesity, and abdominal obesity, associated with low self-efficacy and other metabolic risk factors. Significant differences were found between the Swedish-born and foreign-born participants where the foreign-born had a higher proportion of overweight/obesity as well as lower self-efficacy. Increasing self-efficacy may be a first step towards a healthier lifestyle, and subsequently, a better long-term health outcome, but cultural differences may need to be acknowledged to understand individual perspectives on health. Recognising different distributions of metabolic risk factors by region of birth is essential to improve health equity and develop tailored lifestyle interventions.

### CRediT authorship contribution statement

**Beata Borgström Bolmsjö:** Writing – review & editing, Writing – original draft, Project administration, Data curation, Conceptualization. **Simona Chiarappa:** Writing – review & editing, Writing – original draft, Project administration, Methodology, Formal analysis, Data curation, Conceptualization. **Emelie Stenman:** Writing – review & editing, Writing – original draft, Project administration, Methodology, Formal analysis, Data curation, Conceptualization. **Anton Grundberg:** Writing – review & editing, Writing – original draft, Formal analysis, Conceptualization. **Kristina Sundquist:** Writing – review & editing, Supervision, Project administration, Funding acquisition, Conceptualization.

## Declaration of competing interest

The authors declare that they have no known competing financial interests or personal relationships that could have appeared to influence the work reported in this paper.

## Data Availability

Data will be made available on request.
